# Community healthcare workers’ experiences with the care of clients on antiretroviral therapy in the community

**DOI:** 10.11604/pamj.2022.41.117.28631

**Published:** 2022-02-10

**Authors:** Mygirl Pearl Lowane, Hilda Nwamuhohova Shilubane, Rachel Tsakani Lebese

**Affiliations:** 1 Department of Public Health, Sefako Makgatho Health Sciences University, Pretoria, South Africa,; 2 Department of Advanced Nursing Science, University of Venda, Thohoyandou, South Africa,; 3 Department of Nursing Science, University of Venda, Thohoyandou, South Africa

**Keywords:** Community healthcare worker, HIV programme, experiences, antiretroviral therapy client

## Abstract

**Introduction:**

community healthcare workers are members of the community affiliated with community-based organisations to implement Primary Health Care (PHC) in the district health services of South Africa. Among other roles, they are expected to care for clients on antiretroviral therapy (ART) in the community. The purpose of this study was to explore how community healthcare workers describe their experience regarding the management of ART clients in the community environment.

**Methods:**

a qualitative phenomenological approach was used. Semistructured focus group discussion, observations, and document analysis were conducted with 39 community healthcare workers who had ART clients in their register. Inductive coding was used to determine invariant constituents, reduce constituents to categories, and cluster categories into themes. Reliability and validity were accomplished through intercoder agreement, audio recording, triangulation, bracketing, and member checking.

**Results:**

results identified five core themes related to what community healthcare workers experience during the management of HIV clients in the community environment. They explained a wide-ranging insight into their experiences in providing care to ART clients. They described a number of concerning situations that included meaningful roles, feelings about the clients´ engagement, clients´ improper linkage to care, perceived barriers and perceived influences.

**Conclusion:**

several challenges were encountered by community healthcare workers while implementing the HIV programme in the community. The study saw the need for health departmental awareness for community acceptance of community healthcare workers work. This will thus impact positively in community healthcare workers programmes and thus improving the provision of primary health care services particularly in HIV programme.

## Introduction

Community Healthcare Workers (CHWs) are members of the community enrolled in the programme under the Non-Profit Organisation (NPO) or Department of Health (DoH) on the contract bases receiving stipend monthly [1]. The CHWs affiliated with community-based organisations are central to the implementation of Primary Health Care (PHC) in the district health services of South Africa [2].

These CHWs are expected to provide comprehensive and patient-centred primary healthcare services that will enable universal caring to the clients outside the health facilities [3]. They are trusted frontline health workers who have a close understanding of the community they serve and can be valuable contributors to patient-centred culturally sensitive care [4]. Meaning that their main responsibility is to care for clients referred to the community from respective healthcare facilities. Furthermore, they are referred as paraprofessionals after completion of a two-year training programme [3]. Other authors described paraprofessional as an individual with some form of secondary education and subsequent formal training lasting a few months to more than a year [5].

Community Healthcare Workers play vital roles in increasing coverage of basic health services and they also have various obligations which include linking the community with Primary Health Care (PHC) services and supervising chronic clients in the community [2]. The presence of CHWs bridge the healthcare equity gap by complementing an overstretched healthcare workforce and thereby increasing the availability of basic health services especially in hard-to-reach areas [5]. Though the history of CHWs was traced back to 1970s, their role was primarily to improve maternal and child health, and to manage common infectious diseases where there were limited healthcare manpower and low access to basic health services [1]. The use of CHWs is one of the strategies to address the growing shortage of health workers, particularly in low-income countries [3,6,7]. However, the expansion of utilizing CHWs to meet population needs is also noted even in high-income countries. Furthermore [4] mentioned that CHWs performed common and clinic-specific roles such as outreach, health education and coaching, community resource linkage and facilitating communication between clinician and patient. They also play an important role in fighting the pandemic, especially in countries with less resilient healthcare systems [8].

Community healthcare workers perform multiple functions such as participation in Human Immunodeficiency Virus (HIV) programme. Amongst other things in HIV programmes, they are expected to refer community members for HIV testing, linking them to care, monitoring those referred, accompanying them to clinic appointments, providing psychosocial support, conduct household visits and making referrals to other services [9]. The main goal on HIV programmes was to sustain clients´ adherence to antiretroviral therapy, to prevent lost to follow-up, missing of clinic appointments and regress. The CHWs are deployed around South Africa´s primary healthcare clinics, to offer indispensable support for the world´s largest HIV/AIDS treatment programme [10].

Notedly in sub-Saharan Africa (SSA) the provision of antiretroviral therapy has multiple challenges, including the weak healthcare systems and attrition of ART clients. Hence best solution to overcome these challenges was to engage CHWs [11]. Many studies have been conducted on the roles CHWs play in the community and little is known about their experiences in providing those roles. Hence, this study´s main objective was to explore the experiences of the CHWs in caring ART clients.

## Methods

**Overview and design:** the methodology undertaken for this study was a qualitative phenomenological approach that involved five focus group discussions with CHWs using an interview protocol. The phenomenological approach was considered because it focuses on the lived experiences of a phenomenon, which in this case is the experience of caring the ART clients in the community [12]. This approach views the methodology as a series of logical steps, accounts for multiple perspectives from CHWs, and utilizes rigorous data collection and analysis [12-14]. In this study, a lengthy and rigorous analytic process, and multiple validity and reliability approaches were used based on this belief system.

**Sampling and CHWs:** purposive criterion sampling was used to identify CHWs who have experienced the phenomenon of caring ART clients in the community. This method of sampling helps to create a homogenous sample of CHWs that have all experienced the phenomenon. CHWs were selected from five districts based on role in the community in order to maintain homogeneity of the sample. They were selected because they had cared ART clients. Participants were eligible for inclusion if they had one-year experience caring for ART clients and resided in the selected districts. Each focus group, one per district comprised of an average of seven CHWs, and a total of 39 CHWs participated in the study. Although there is no standard for a maximum number of CHWs in qualitative research because of its nature, the researchers in this study have identified a sample size of 39 to be adequate. In this study, CHWs were no longer recruited when data saturation was attained. Implying that there was no new information provided to add in the understanding of the phenomenon.

**Data collection:** CHWs were contacted personally with an ethical clearance letter, permission to conduct the study and consent form explaining their rights as CHWs. Written consent was obtained from each participant and assigned a pseudonym. The first author, who completed data collection, was trained in qualitative methodology and bracketed biases before beginning data collection to assure data accuracy. Focus group discussions using semi structured guide (Annex 1) were conducted with CHWs privately in their organisation boardroom. A semi structured interview guide was developed, reviewed by co-authors, and edited based on feedback. All focus group discussion (FGDs) were audiotaped for accuracy. The interview protocol included seven questions under the umbrella of the main objective, namely: to explore the experiences of CHWs in caring ART clients (Annex 1). Throughout the process of the interviews, probes and follow-up questions were added as needed to encourage elaboration and clarify responses. Specific questions were added as the interview process progressed in response to developing themes. Immediately after each interview, verbatim transcripts were generated by the first author who conducted the focus group discussions. Data collection was completed over a period of four months and ended upon saturation of the data, when no further themes or new information emerged to add to the understanding of the phenomenon.

**Data analysis:** inductive data analysis was used in this study. Once text has been prepared to individual transcript, close reading of transcripts was done to immerse the researcher in the data. All transcripts were read again and memos were recorded to further immerse the researcher and highlighted key concepts until the researcher was familiar with its content and gained an understanding of the themes and events covered in the text [15,16]. Themes were identified by the researcher. Within each theme, search for sub-topics and selection of appropriate quotations that convey the core theme were conducted. Reduction and elimination of statements that were not describing the experience of CWHs was performed to determine the variant constituent of the experiences. This process involved asking whether the statement contained a moment that was necessary for understanding the experience and whether it could be labelled. Clustering was performed to group related constituents together, and each category was given a thematic label. This step was repeated several times to further group and reduce categories until all constituents were clustered and reduced into five core themes.

**Reliability and validity:** reliability techniques used in this study include cording of detailed field notes, an audio recorder for accuracy, and intercoder agreement from other authors. An independent coder was also consulted. There after met with the first author to discuss codes and agreement reached. All the significant discrepancies, and any small differences were discussed and resolved to create one set of themes. Data source triangulation to collaborate evidence, bracketing to clarify bias, and member checking was conducted with CHWs to determine the credibility of the findings and interpretations. Appointments were made with the groups to review the final themes, as well as a sample of the in variant constituents of those themes. CHWs were asked to examine these themes and reflect on the accuracy. CHWs reflected accuracy on their perceptions and experiences.

**Ethical approval and consent to participation:** the ethical clearance for this study (SHS/17/PH/13/1608) was granted by the University of Venda, Research Ethics Committee. The permission to collect data in the healthcare facilities and the non-government organisation was obtained from the Department of Health, Limpopo Province. Furthermore, permission from DoH was extended to district executive managers and the mangers of NGOs for permission to interview their cadres. The aim of the study was specified to the CHWs concerned and informed consents were requested and obtained voluntarily.

## Results

A total of 39 CHWs participated in the study. Of the 39 CHWs 31 were females and 8 were males. Most CHWs´ ages range from 25-49 years but four were older than 50 years. Of the participating CHWs, 16 had 6-10 years´ experience, nine had 11-19 years´ experience, five had at least 20 years´ experience while nine had up to five years´ experience in their current organisations. Of the 39 participating CHWs, 19 had never received training for providing home-based care but had received in-service training provided by their organisations, and 20 had received formal training in community home-based care. Only 18 CHWs were trained in the management of HIV-positive clients in the community, while 15 had received adherence counselling training. Five themes emerged from the experience of CHWs in this study, including (1) meaningful role, (2) feelings about the clients´ engagement, (3) improper linkage to care, (4) perceived barriers, and (5) perceived influences.

**Meaningful roles:** community healthcare workers experienced community caring through a variety of roles. The most primarily roles mentioned involves conducting home visits, health education, direct observed treatment support, promoting treatment adherence and counselling, identifying clients in need of care and link them with the clinic and advocate for social grant support. The CHWs expresses that their roles were meaningful for ART clients in the community: *"Some clients and family members acknowledge the work that we are doing for them. Some people call you while you visit others, asking you to come to their houses."*(FGD B6). *“One client asked me to visit and help S”, because she noticed that she was drastically lost weight. She even mentioned that she tried to go to the clinic, but failed, hence she needed me to help her.”* (FGD A5).

However, a few CHWs mentioned that they would go extra mile if they had resources and more support from nursing staff. They mentioned that some areas need transport to reach and accompaniment by clinic sister as they are unable to tell whether the condition require clinic intervention or not. Some felt that more efforts needed to be made with community HIV management program in a broader sense rather than just their individual role: *“Most HIV clients default treatment and become lost to follow up in the community. I don´t think we are doing enough to educate the society what that means and what are the consequences. We are doing more on educating the clients, than the society.”* (FGD C1).

**Feelings about the clients´ engagement:** all CHWs felt that their involvement was important for their clients. This is what CHWs had to say: *“At first it was difficult and I felt uncomfortable with the subject of HIV, because we use to care for older clients with diabetics and high blood... then after I realised that HIV condition is just as important or more important like any other chronic conditions.”* (FGD C4). CHWs felt they had a same responsibility to help their ART clients to shape their behaviour while living with HIV the same as with the clients with other chronic diseases. Many CHWs also expressed importance in terms of the ART clients´ future, because they were helped to remain in care and adhering to ART. Other CHWs felt that it was very essential to engage with HIV clients because they believe that it form the foundation of safe lifestyle habits to their clients. During the interview they displayed an enjoyment and high level confident with engagement of these clients. One CHWs mentioned the following: *“This is one area I enjoy so much when supporting my clients, because I am able to allay their anxiety and do counselling when I realised that they start to worry”. most of them complain about the burden of taking the medication for the rest of their life, while others worried about the relationships”., with the training and experience acquired, it is simpler to me to talk to them.”* (FGD D3).

Majority of them felt appropriateness to help the ART clients to cope with the new living situation for their well-being. However, they felt that the home environment was not conducive for the ART clients´ counselling session as compere to clinic environment where they [ART clients] provided with more information about the progress of their condition. Furthermore, CHWs expressed that HIV counselling was important, but they worry about the amount of time spent with the clients, hence they reported that it was minimal as the subject of ARVs is broad and it need to be executed accordingly. Although some CHWs did express that they would like to dedicate more time with ART clients, other did not feel that it is necessary, as most of their clients are coping well with the condition. “*I mean honestly, not all clients are still having challenges with taking their [ART clients] medication, in facts we need to win them gradually and concentrate to those in real need of our care. I can´t see myself spending more time with the person who knows his subject matter.”* (FGD E2).

**Improper linkage to care:** during the FGDs, the CHWs mentioned that a poor referral system and improper linkage to care posed serious challenges to the management of ART clients in the community. All ART clients should be allocated to specific CHWs for further management in the community. When the researcher asked how the clients were referred and linked to the CHWs´ care, the following answers were provided by the CHWs: *“There is no actual linkage to care from the clinic to us in the community. We just identify the clients when we do home visits and then register them in our register.”* (FGD A5), *“...there is a serious challenge because we don´t report to the clinic on a daily basis. We only go there once a month during the stakeholder meeting...., clients are just told to expect a visitor who will be checking them for compliance without me being notified. We only find the clients ourselves during household visits. Then we register them in the carer´s profile register.”* (FGD C7).

On the contrary, some CHWs stated the following: *“Every day in the morning, we report at the clinic and sign the time register before going out. After that, we ask the data capture to give us the list of clients who were initiated a day before and even during the weekend. So, we decide among us who will be caring [for] who looking at the geographic area. After that, we go and meet the client and do self-introduction, [explaining the] main purpose for the visit and our role to him/her.”* (FGD D8).

Participating CHWs were further asked how clients linked to their care. The researcher wanted to establish whether there are formal written documents for referrals, or any treatment care plans discussed with them. The responses were as follows: *“There are no formal referral letters. Nurses sometimes tell us verbally that we need to visit so and so. No treatment plan or instructions on how we should care (for) that client. We just go and visit them like any other client and check for side-effects and whether the client has taken the treatment. Only that.”* (FGD C2). *“Nurses do not allocate the clients for you. Hence there is no referral letter or care plan. We only manage the clients based on our experience acquired during the service. That is why [it] is very difficult to know what is it that we should achieve while monitoring the client.”* (FGD B4).

A probing question was asked on how the CHWs was able to identify the clients in need of care in the community while there is no proper linkage to care from the clinic. Majority described how they manage to get those clients [ART clients] during home visits. Following is one of the responses: *“I only knew about”, after I have been called by one of the family members and found the client was in the critical state.”* (FGD D3).

Majority of CHWs voices that it is difficult and challenging to approach a client based on the rumours or referred by another client. Because when divulging the information to that client about who told you about his/her illness, other clients acknowledge; but majority displays anger. As a result, some CHWs were hesitant about how to communicate with clients or family members concerning wellness and had a difficult time gauging between enrolling the client in their care and overstepping their role.

**Perceived barriers:** community healthcare workers voiced various barriers to ART programme, the strongest of which were non-disclosure or disclosure with false condition and denied access by ART clients and family members.

**Non-disclosure or disclosure with false condition:** community healthcare workers were faced with the challenge of non-disclosure or false disclosure when they conduct support visits to ART clients. Non-disclosure hinders treatment and affects adherence to ART. The interviewed CHWs mentioned that clients hide their HIV-positive status from their family members, and as a result, their family members could not encourage regular ART clinic visits. Most CHWs participating in the current study´s FGDs experienced the same challenge when visiting the clients. During the FGDs, they shared the following experiences: *“I visited one client who had reportedly missed three consecutive visits, and when I arrived, I found the whole family seated outside and they were enjoying their tea. I greeted them and told them that I was there to see S…, the client concerned quickly replied that they are all fine. No one is having TB or HIV in the family. Her mother added by saying S..., has been healed from the treatment of ulcer received from the clinic. I then realised that S…, did not disclose the status. I requested the phone numbers of S...., so that I can call or send the message that she is wanted at the clinic, but she refused. Then I left but told S…, if she gets a chance, she must go to the clinic for a follow-up visit.”* (FGD C2).

One CHW, FGB E5 mentioned that he has been warned by the ART client not to come to his home because his wife does not know his status. Similar challenges were also reported by other participants (CHWs) who indicated that some family members or clients themselves stated a different diagnosis. Some CHWs revealed that most of the conditions the clients and/or the family members claimed to have, were diabetes, hypertension and peptic ulcers. Apart from medical conditions, other participants (CHWs) indicated that some clients implied that they were bewitched while others said that they were possessed by demons explaining why they were very sick (FGD E5). Other comment from the participant is as follows: *“I went to support D… and I did not find her. When I ask her mother about her whereabouts, she responded that she went to the clinic to collect medication for headaches. I further extended my question by asking does she go to the clinic every month, the mother replied that she was supposed to go every month. The mother added by telling the participant that the nurses at the clinic told D..., that there is a blood clot in her brain that is why she has to collect treatment for headache monthly.”* (FGD A4).

**Clients´ and family members´ denial of access for support:** in this situation, both the family members and clients were aware of the clients´ HIV-positive status, but they do not want any CHW near their home, because they believe community will conclude about their status. During the FGDs in the current study, one participating CHW (FGD A6) mentioned that CHWs were associated with TB and HIV by community members. Another participant (FGD A1) added that ‘the community members when they see CHWs visiting a household, they jump to the conclusion that the family visited is affected by HIV or TB´. Participants (CHWs) mentioned that there was a stigma attached to them because of the nature of the work they do. Some community members believed that all CHWs were HIV-positive. In particular, CHWs felt high levels of stigmatisation of HIV was a hindrance to their work, and contributed to the community lack of trust, as their work was often associated with nursing those living with HIV. Many reported that the biggest fear for clients was that of being gossiped about within the community, that people would point fingers at them, saying that they could have HIV if they were seen to be visited by a CHW.

Some responses included the following: *“Yoo!!! When they see us coming, they close the house and when you go there and knock, they keep quiet or sometimes they will answer while in the house stating that Z…, is not at home, meanwhile you saw her while coming.”* (FGD A1).

**Perceived influences:** community healthcare workers experienced success through a triage interaction of positive influences between themselves, their ART clients, and the ART programme. Many CHWs expressed enjoyment for the programme due to their ART clients´ excitement and positive attitude they are getting from them [ART clients]. Most CHWs mentioned that they are super excited more especially when their clients are doing well and following the prescription. Participants demonstrated this positive attitude toward CHWs programme during the discussion through their body language and expressions. In turn, CHWs perceived themselves as very influential figures for those ART clients. one participant expressed: *“Most clients trust us and believe everything we are teaching them, they [ART clients] say they look up to us as their nurse model..., I think that has gone beyond influential for them.”* (FGD D2).

Furthermore, most CHWs believed that the role they are playing have positively impacted to their clients regardless of the barriers and challenges experienced. However, fewer of them felt that they were not significantly influential to their clients, as sometimes they fail to meet their [ART client] expectations due to knowledge gap: *“I know now that a lot of clients under my care do look up to me, but at the same time, it´s not as in like I am the hero in the management of their condition... I think it´s their [ART clients] determination, self-motivation and commitment, hence they are so calm with their condition.”* (FGD E3).

Community healthcare workers believed that the combination of ART management programme and the positive influence of clients was related to their knowledge, confidence and commitment. Their main goal of supporting the clients was to improve the behaviour of their client on ART. The most commonly perceived improvements in behaviour included taking medication on time, regular clinic visits and maintain the health status. Some CHWs strongly believed that even that gradually behaviour changes were very important in ART clients, as most of them reap the benefits of being in the CHWs´ programme.

## Discussion

This study explored the experiences of the CHWs regarding the care of ART clients. The CHWs were increasingly used to provide HIV services and that there was evidence that they play an important role in improving HIV programmes through various community engagements [17]. Exploration with CHWs revealed complex feelings toward CHWs programme that were inconsistent. However, CHWs expressed and demonstrated enjoyment and commitment to the programme. One theme that emerged from this research was CHWs´ roles in community caring. Community healthcare workers perceived that they played many roles in the care of ART patients in the community which is supported by previous research that CHWs perceive themselves as the carers and educators of ART clients and their families [18]. The roles of CHWs bring about changes in behaviour in the community and helping community members on their journey through empowerment [7]. However, some literature highlighted that the role played by CHWs are not necessarily effective because in some instances they are not fully integrated into the health system [17]. In contrast, some literature argued that CHWs perceived their activities as beneficial to the community and it served as a means of motivation for them to remain in the programme, despite challenges, such as their employment uncertainty, remuneration, training, management, supervision and retention [19].

Importantly, the extent to which CHWs feel their service has met community members´ expectations as well as fulfilled their personal needs, their perceptions of their work and their perceptions of other people´s reactions to their work are all key factors of a sense of self-efficacy and self-confidence [20]. Clients´ own sense of enjoyment increased the CHWs´ sense of enjoyment emphasising the need for ongoing engagement. Confidence, on the other hand, improved when the high number of clients comply to the set rules of the programme. Based on the current study´s participating CHWs´ responses, ART clients are not properly linked or referred to CHWs from the clinics. Referral constitutes the handing over of care from one caregiver to another [21]. To ensure a high-quality, sustained treatment process, a referral letter should contain all the client´s necessary information and be shared among other members caring for the client [21]. Clients´ care plan is crucial to assist even CHWs who received little training or no training, as identified in the CHWs demographic profile about the management of ART clients. The CHWs in the current study believe that poor referrals and clinic-community linkages impact negatively on adults´ adherence to their ART appointments because they were unable to monitor compliance of those clients of whom the CHWs were unaware. Strengthening linkages between the community and CHWs as well as clarifying their roles is needed to address the compatibility of the programme.

The findings of this study depicted several barriers experienced by CHWs during community caring. Such barriers include victimisation, discriminatory attitudes, however, the one rank most were the issue of disclosures and denied access. Community healthcare workers in this study were concerned about non-disclosure and false disclosure of their clients´ [ART clients] conditions to their family members. The issue of non-disclosure affects not only a chance to be supported but also the clinical outcomes [22,23]. Some literature revealed that non-disclosure is a criminal act in some countries [24,25]. The criminalisation of HIV non-disclosure has been shown to represent a structural barrier to the healthcare engagement [24]. Hence CHWs sometimes failed to execute their role to people living with HIV in the community.

The CHWs who participated in the current study found it challenging to supervise clients who did not disclose their conditions to family members as they would not access them. This could mean that ART clients who forget to take their ARVs and miss clinic appointments won´t receive the necessary support they need. Research demonstrates that CHWs are uniquely positioned to improve clients´ health outcomes, and the counselling ART clients and family members receive during home visits benefit them all [26]. In the current study, it was not possible to counsel some ART clients and their family members as a result of non-disclosure. Community members might not understand the role of the CHWs, hence, for CHWs to be effective in health promotion and prevention efforts, a shared culture with their communities is critical [27]. However, it was unclear as to which cultural and social elements should be shared for CHWs to be effective [26]. Also, personal characteristics impact a CHW's ability to build trust and rapport with a community member. A few studies evaluated the relative importance of interpersonal relationships between community members and CHWs and conclude that some studies established that socio-cultural characteristic of CHWs had limited significance, but trust was most essential [26]. It could be possible that some ART clients did not welcome CHWs in their homes because they don´t trust them.

From the observations on the finding of the current study, families may be a barrier that contributes to their family member´s unacceptable conduct. Some CHWs believed that families knew about their family member´s condition. Hence it is empirical to involve family members on the subject of community nursing so that they are aware of the CHWs´ roles. Community healthcare workers believed that their role is important to the lives of people living with HIV in the community, hence they experienced joy and excitement. CHWs perceived that their roles in interactive HIV programme yield positively influence on the ART clients.

**Limitations:** this study was qualitative in nature, conducted in selected rural South African setting. Participants may have reported information that are personal experienced to present themselves in the best possible light which may result to social desirability bias. The sample is not necessarily a representation of all the population of CHWs in the area, hence the findings cannot be generalised to other CHWs´ experiences.

## Conclusion

The study was conducted with the aim of exploring the experiences of CHWs regarding the care of ART client in the community environment. The study highlighted several challenges encountered while implementing the HIV programme in the community. The challenges experiences included poor linkage to care, disclosure and discriminatory attitudes, where they are sometimes denied access to the households. The inherent tensions in their work associated with caring for stigmatised conditions such as HIV need to be addressed. Although CHWs face several challenges while executing their roles, they remained excited when assisting ART clients in the community. The study portrays that there is a need for health departmental awareness for community acceptance of CHWs work. This will thus impact positively in CHWs programmes and thus improving the provision of primary health care services particularly in HIV programme. Efforts in this regard must be made to allay community fears and explain the value of household-level support visits by CHWs that form part of their formalised roles. As the healthcare system continues to integrate CHWs formally in their programmes, they should acknowledge the CHWs´ roles and develop support system policies for them.

### What is known about this topic

- Community healthcare workers are cadres who did not receive formal nursing training but expected to conduct some of the nursing care on the clients in the community.

### What this study adds

- The community healthcare workers (CHWs) play many roles in the community for example, caring and educating ART clients and their families, thus increased the CHWs´ sense of enjoyment and confidence when the high number of clients comply to the set rules of the HIV programme;
- Poor linkage or referred of ART clients to CHWs from the clinics interferes with high-quality sustained treatment process;
- Barriers such as victimization, discriminatory attitudes, nondisclosure and false disclosure of the client´s conditions to their family members and denied access to their home hinders with duty of CHWs, as result mortality and morbidity rate in the community are not accurately registered in the healthcare system surveillance.

## Annex

**Annex 1**: interview guide for community health workers (PDF-89kb)
